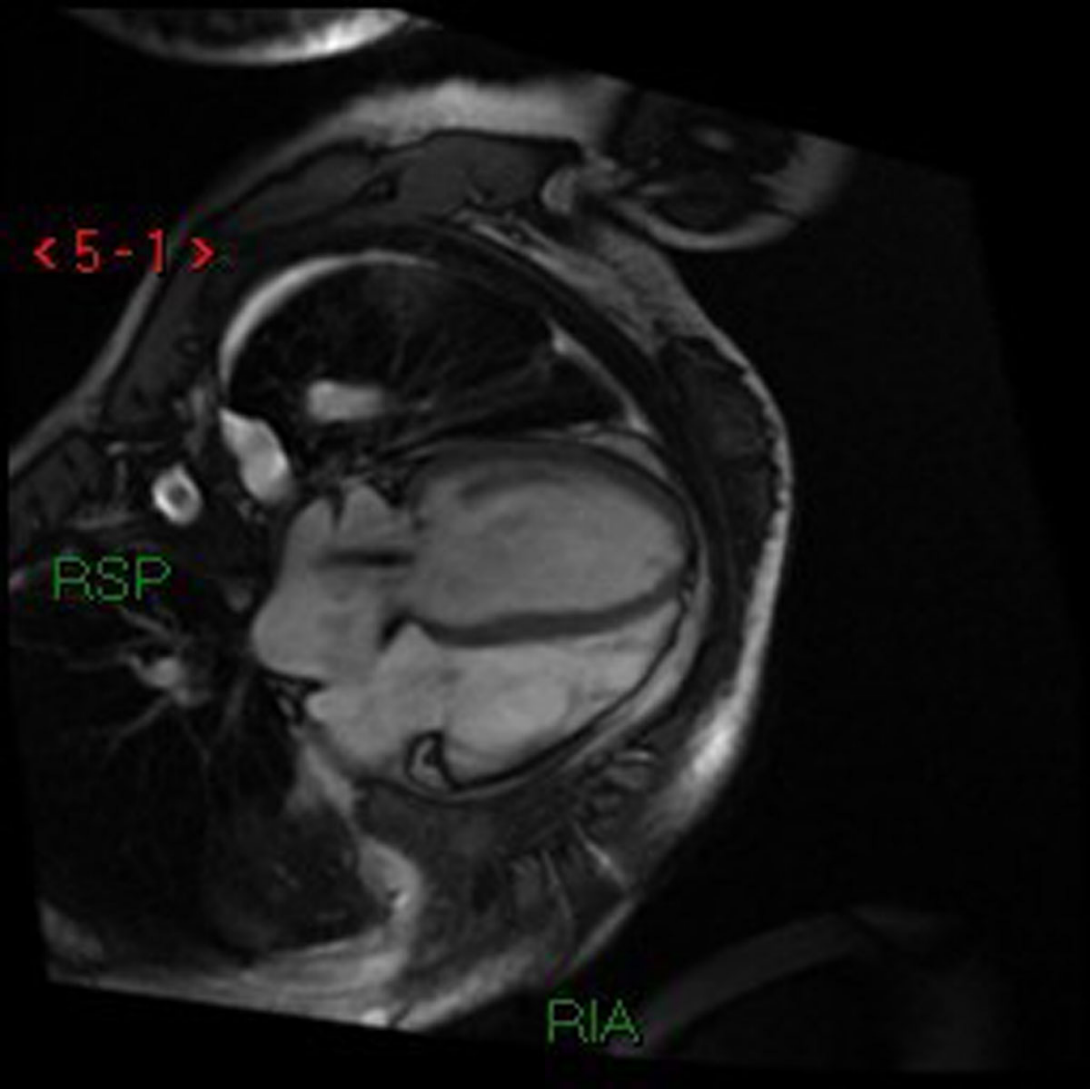# Valvular disease in patients with active sle and clinical suspicion of acute myocarditis : assesment by cardiovascular magnetic resonance

**DOI:** 10.1186/1532-429X-17-S1-P298

**Published:** 2015-02-03

**Authors:** Cielmar G Abarca

**Affiliations:** 1 Cardiovascular Imaging, National Institute of Nutricion and Medical Sciences "Salvador Zuiran", Mexico

## Background

Systemic lupus erythematosus (SLE) is a disease of autoinmune origin that may involve numerous internal organs of the body. It is associated with high cardiovascular morbility and mortality. The cardiovascular magnetic resonance (CMR) provides us with a global evaluation of patients with active SLE and clinical suspicion of acute myocarditis. OBJETIVES The aim of study was the identification of cardiac alterations in patients with active SLE and clinical suspicion of acute myocarditis.

## Methods

A total of twenty - eight patients were included in the study, with an average age of 30.7 years ( SD 12.7 yrs), 96% female, diagnosed by criteria of American College or Rheumatology (ACR). Cardiovascular magnetic resonance (CMR) was performed on a 1.5 T scanner and include the following sequences: steady- state free precession, STIR T2 weighted (Ts-2W), T1- Weighted spin - echo before and after gadolinium injection and late enhancement.

## Results

The ejection fraction of left ventricle < 50% was found in 54%, 83.4 % with contractility alterations, 87.5% with valvulitis and affection of the subvalvular apparatus in 50%, pericardial effusion was observed in 62.5% and tissue characterization suggestive of myocarditis for the Lake Louise criteria were found to have global and relative enhancement in 37.7 and 50 % respectively and late gadolinium enhancement (LGE) in 66.7%.

## Conclusions

In our study group we found that patients with acute myocarditis asoociated with SLE have a high incidence of valvulitis and affection of the subvalvar apparatus.

**Figure 1:**
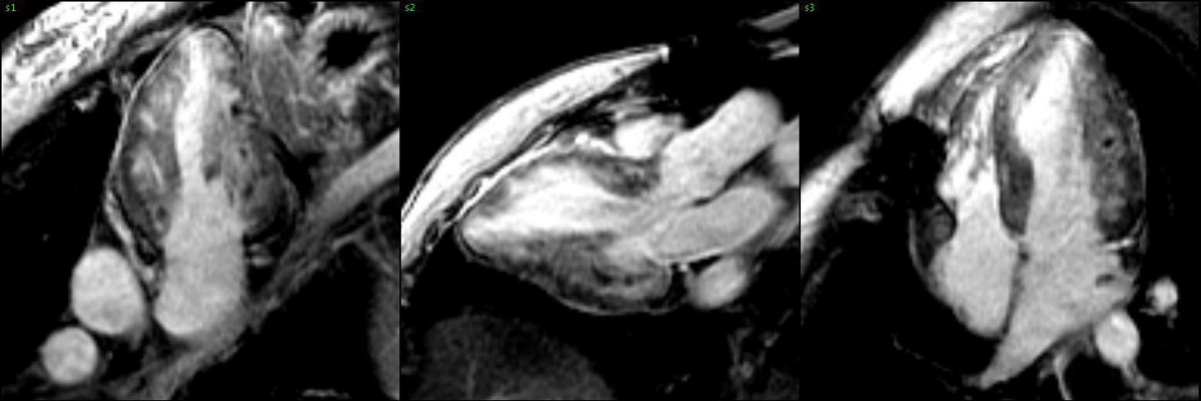


**Figure 2:**